# Medical Brain Drain in Albania: Migration Attitudes Among Medical and Nursing Students

**DOI:** 10.3390/nursrep15080264

**Published:** 2025-07-22

**Authors:** Vasilika Prifti, Denada Selfo, Aurela Saliaj, Sonila Qirko, Emirjona Kicaj, Rudina Çerçizaj, Juljana Xhindoli, Liliana Marcela Rogozea

**Affiliations:** 1 Faculty of Medicine, Transilvania University of Brasov, 500036 Brasov, Romania; sonila.qirko@unitbv.ro (S.Q.); emirjona.kicaj@unitbv.ro (E.K.); rudina.cercizaj@unitbv.ro (R.Ç.); r_liliana@unitbv.ro (L.M.R.); 2 Faculty of Health, University of Vlora, 9401 Vlora, Albania; denada.selfo@univlora.edu.al (D.S.); aurela.dai@univlora.edu.al (A.S.); juljana.xhindoli@univlora.edu.al (J.X.)

**Keywords:** brain drain, migration, nursing students, medical students, healthcare workforce, Albania

## Abstract

**Background**: The migration of healthcare professionals poses a serious threat to health systems worldwide. This study examines attitudes toward brain drain and the factors influencing migration tendencies among medical and nursing students in Albania, with particular attention to nursing workforce implications. **Methods**: A cross-sectional study was conducted with 610 students in the 2024–2025 academic year using the 16-item Brain Drain Attitude Scale (BDAS). Socio-demographic and academic data were also collected. **Results**: The mean BDAS score was 53.43 ± 16.88. Pull factors (mean: 40.25 ± 12.76) were stronger motivators than push factors (mean: 13.19 ± 4.13). A total of 487 nursing, 73 midwifery-nursing, and 50 medical students participated (95% response rate). Nearly 40% expressed a desire to work abroad, citing financial prospects (48.2%), better living standards (46%), and personal freedom (42.1%) as reasons. Higher migration tendencies were seen in females (β = 0.50, *p* = 0.049), medical students (β = 1.01, *p* = 0.001), and third-year students (β = 0.46, *p* = 0.011). **Conclusions**: Migration tendencies are high among future Albanian healthcare professionals, with significant implications for the nursing workforce. Targeted policies are urgently needed to address brain drain through workforce investment and retention strategies.

## 1. Introduction

Medical brain drain is a global phenomenon that has detrimental effects on the healthcare systems of developing nations. It is estimated that more than 10% of medical graduates from developing countries like Albania emigrate due to better wages, work conditions, and training opportunities available in high-income Western countries. This is particularly concerning for nurses and midwives, whose workforce density is 30–50% lower than that of physicians in many developing countries [1]. A recent study assessed the willingness of 1505 undergraduate students to go abroad for higher studies and residency. It showed that most nursing and public health students intending to migrate come from rural parts of the country, motivated by high salary prospects abroad and poor working conditions at home [2]. The issue of medical brain drain is not a new one, and it is similar to most phenomena taking place in developing countries such as the Western Balkan Countries [3]. Migration is becoming a phenomenon of global dimensions insofar as it concerns hundreds of millions of people in the entire world. The first reports on medical brain drain date back to the seventies. The movement of healthcare professionals to other countries is increasingly recognized as a major concern globally and among the 28 countries of World Health Organization (WHO) European Region. A significant part of this phenomenon has been the migration of healthcare workers from the less developed countries to the more developed countries, known as the South-to-North or medical brain drain. In the WHO European Region, medical brain drain is particularly pronounced for some of Eastern European countries, among them Albania [4]. Young people in Albania aspire to leave the country [5].

The purpose of this study is to examine the migration attitudes and intentions of medical and nursing students in Albania. Understanding these perspectives is crucial for workforce planning and policy development, particularly given the increasing severity of the situation. To the best of the authors’ knowledge, no prior research has specifically explored the migration motivations and attitudes of current medical and nursing students in the Albanian context. This study seeks to fill this gap by (a) providing a descriptive analysis of the international migration tendencies among future healthcare professionals and (b) identifying key push and pull factors that influence their decision to migrate. By gaining deeper insights into these motivations, the study aims to contribute to the development of policies that address the challenges associated with medical brain drain.

### 1.1. Literature Review

The push–pull framework is one of the most widely accepted models for understanding international migration, including among healthcare professionals. Push factors are negative conditions in the home country that drive individuals to leave, such as low salaries, poor infrastructure, and political instability. In contrast, pull factors are positive attributes of destination countries, such as career opportunities, better wages, and improved quality of life. Several studies in the Western Balkans and beyond have used this model to explain health worker migration trends [3,4]. In the Albanian context, economic hardship and lack of career growth are dominant push factors, while professional advancement and stability abroad function as primary pull forces. The current study builds on this conceptual framework by assessing its relevance among undergraduate medical and nursing students.

### 1.2. Research Questions and Hypotheses

This study is guided by the push–pull theory of migration, which suggests that individuals’ decisions to migrate are influenced by both negative conditions in their home country (push factors) and attractive opportunities abroad (pull factors).

In the context of health workforce migration, push factors may include limited career opportunities, low wages, or poor working conditions, while pull factors often involve better remuneration, professional development, and quality of life abroad. This framework helps explain the complex motivations behind medical and nursing students’ intentions to migrate.

Based on this theoretical framework, the following research questions and hypotheses were formulated:

What are the main attitudes of medical and nursing students in Albania regarding migration?

**H1.**  *Pull factors will score higher than push factors on the BDAS.*

**H2.**  *Medical students will exhibit higher migration tendencies than nursing and midwifery students.*

**H3.**  *Female and third-year students will report stronger migration intent.*

## 2. Materials and Methods

### 2.1. Study Design

This cross-sectional research was conducted between November and December 2024.

### 2.2. Study Setting and Participants

The sample included 487 nursing students, 73 midwifery-nursing students, and 50 medical students. The response rate was 95%, and participants were selected through simple random sampling from universities located in three different Albanian regions. Participants were recruited from public and private universities across three Albanian regions, selected to reflect geographic diversity. While national representativeness was not the primary aim, efforts were made to ensure coverage of different institutional settings.

Inclusion Criteria: Nursing and medical students, aged over 18 years, conscious and oriented to time and space, no visual, audial, or lingual disabilities, and no mental disorder, being a volunteer to participate in the study.

### 2.3. Instrument and Data Collection

The data were collected using a questionnaire containing socio-demographic features form and the Brain Drain Attitude Scale (BDAS). Originally developed in Turkish by Öncü et al. (2018), the BDAS was translated into Albanian for this study, with the original validation reporting a Cronbach’s alpha of 0.91 [6,7].

The scale aims to determine the attitudes of nursing students towards migration. The one- dimensional scale of 16 items has two components, which were “pull (1–6,8,10,12,14–16) and push (7,9,11,13) factors”. Pull factors include items such as opportunities for better income, standard of living, and professional development abroad. Push factors include insecurity about the future and dissatisfaction with conditions in the home country. Example pull factor item: “I want to work abroad to earn better income.” Example push factor item: “I feel insecure about my future in my home country.” The BDAS is scored on a 5-point Likert scale, ranging from 1 (Strongly Disagree) to 5 (Strongly Agree). The total score ranges from 16 to 80. Two items within the scale are reverse-coded, meaning their scores should be inverted when calculating the final score: Item 3: “I do not need to go abroad because I have enough career opportunities in my country. And item 15: “I am not interested in news about life abroad.”. A higher score on the scale reflects a more positive attitude toward migration and a greater tendency to migrate, whereas a lower score suggests a negative attitude toward migration and a lower desire to migrate.

### 2.4. Psychometric Properties of the BDAS

#### 2.4.1. Validity and Reliability Analysis

For the 16 questions in the questionnaire, an analysis of validity, reliability, and factor analysis was carried out to determine the structure of the questionnaire.

#### 2.4.2. Translation and Content Validity

Initially, forward–backward translation was performed. The BDAS was translated into Albanian, and both linguistic and conceptual equivalence of the items were confirmed. A back-translation process was carried out to ensure consistency between the Turkish and Albanian versions of the scale. The original scale was translated into Albanian by translators highly proficient in both languages.

A pilot study was conducted in September 2024, involving 10 students, to identify any unclear questions in the scale. The data collected from this pilot study were excluded from the final data analysis. Based on the results of the pilot study, minor adjustments were made to the wording of certain items to enhance their clarity.

#### 2.4.3. Construct Validity

Factor analysis of the Albanian version of the scale yielded a single-factor structure, with factor loadings ranging from 0.735 to 0.951.

#### 2.4.4. Internal Consistency

In this study, the correlation coefficients between the average BDAS item scores ranged from 0.73 to 0.95, indicating a satisfactory level of reliability. Cronbach’s α for the BDAS, calculated to assess internal consistency and uniformity, was 0.846, which is considered high.

#### 2.4.5. Test–Retest Reliability

As a part of the pilot study, the test–retest reliability was evaluated by administering the same questionnaire to a sample of 10 students two weeks apart. The results were consistent and the Albanian version of the BDAS can be regarded as having excellent psychometric properties, closely aligning with those of the original scale.

#### 2.4.6. Bias

The study implemented several measures to address potential sources of bias. Clear inclusion and exclusion criteria ensured a relevant and capable sample, while the BDAS underwent rigorous adaptation and validation, including forward–backward translation, expert review, and pilot testing, to ensure cultural and contextual suitability. A pilot study identified and corrected ambiguities in the questionnaire, improving clarity and reducing response bias. Ethical guidelines were strictly followed to ensure voluntary and unbiased participation. Finally, appropriate statistical methods were employed to analyze the data, ensuring robust and reliable findings.

### 2.5. Study Size

The study included 610 students. The sample size was calculated using the single proportion formula with z = 1.96, assuming 50% migration intent, 4% margin of error, and 95% confidence interval, following Pourhoseingholi et al. (2013) [8].

### 2.6. Ethical Considerations

All ethical guidelines as well as the Helsinki declaration were strictly followed for this study. Before data collection, the research protocol was reviewed and approved from the Faculty of Health, University of Vlore. The purpose, risks, and benefits concerning the research were explained to potential participants, who were also informed that their responses would be confidential and anonymous, that participation was voluntary and that they could withdraw at any point.

### 2.7. Statistical Analysis

The data analysis was conducted using IBM SPSS Statistics version 25 (IBM SPSS Corp., Armonk, NY, USA). Descriptive statistics, including mean, standard deviation, median, frequency, minimum, and maximum values, were calculated for all variables. The Kolmogorov–Smirnov test was applied to assess the normality of distribution for continuous variables. Non-parametric tests were used for data that did not meet normality assumptions. The Mann–Whitney U test was utilized for two-group comparisons, whereas the Kruskal–Wallis test was employed for comparing three or more groups. Additionally, logistic regression analysis was performed to determine factors influencing attitudes toward brain drain. Two separate linear regression models were performed to assess the association between the mean BDAS score (as a measure of emigration intentions) and push and pull factors. A *p*-value of less than 0.05 was considered statistically significant.

## 3. Results

The mean age of the students was 20.61 ± 2.17 years. The demographic and educational profile highlights a predominantly young, single, and female student population, primarily engaged in nursing studies at the undergraduate level across various universities in Albania (Table 1). In this study, a ‘young person’ is defined as an individual between the ages of 18 and 30, consistent with definitions used by the United Nations and WHO for youth and young adults in health and education research (World Health Organization, 2014; United Nations, 2013) [9,10].

Of the participants, 60% chose to study medicine or nursing due to a desire to help people in need, 15% cited migration opportunities as a key factor in their decision, 12% reported being influenced by family members, 8% pursued these studies primarily to obtain a degree and 5% mentioned other reasons for their choice.

The responses to the Brain Drain Attitude Scale highlight that students are primarily influenced by pull factors, which make migration appealing, rather than push factors, which drive them away from their home country. The most prominent reason for wanting to migrate is financial opportunity, with almost half of the respondents (48.2%) stating that they would like to work abroad to earn more money. Similarly, 45.9% believe that working abroad would improve their standard of living, and 42.6% are influenced by job advertisements for employment opportunities in other countries. Many students (39.5%) think that living abroad would make their life easier, while 38.5% feel that remaining in their home country is a waste of time. Additionally, 37.7% believe they would have a more satisfying professional life abroad, and 32.0% believe that working abroad would make them happier. 33.9% of the students indicate that they are willing to endure hardships to secure employment abroad, demonstrating a strong commitment to migration despite potential challenges.

While pull factors dominate migration attitudes, push factors also play a significant role. Concerns about safety and freedom are among the most influential reasons to leave the home country, with 42.1% of respondents expressing a desire to live in a country that offers greater freedom of thought and personal security. Additionally, 40.2% would prefer to work in a country where they do not have to worry about their future. Political conditions also contribute to migration tendencies, as 31.6% of students indicate that they feel pressured by the political environment in their home country. Table 2 outlines variations in emigration attitudes across participant subgroups.

The mean scores on the Brain Drain Attitude Scale, analyzing pull factors, push factors, and the total score across different demographic and educational characteristics, are shown in Table 2.

The field of study was the only variable significantly associated with migration attitudes, with *p* = 0.002 for pull factors and *p* = 0.004 for the total BDAS score. Medical students reported significantly higher brain drain attitude scores compared to nursing and midwifery-nursing students. Other variables, including gender, age, marital status, level of study, and year of study, did not show statistically significant differences (*p* > 0.05).

Regression analysis showed that being female (β = 0.50, ***p*** = 0.049), a medical student (β = 1.01, ***p*** = 0.001), or in the third year of study (β = 0.46, ***p*** = 0.011) significantly predicted higher BDAS scores. These variables were positively associated with stronger migration intentions.

To examine the association between emigration intention and the core dimensions of the push–pull model, two separate logistic regression models were conducted. In the first model, which included only push factors as predictors, results indicated a strong and statistically significant association with emigration intention (β = 3.97, SE = 0.32, ***p*** < 0.001, 95% CI: 3.33–4.60). This suggests that higher dissatisfaction with local conditions—including limited professional development opportunities and inadequate support systems—correlates with a greater likelihood of intending to migrate.

In the second model, focused on pull factors, results again demonstrated a significant relationship (β = 7.44, SE = 0.66, ***p*** < 0.001, 95% CI: 6.15–8.74), indicating that favorable perceptions of working abroad—such as better financial opportunities, improved training, and career advancement—substantially increase the odds of migration intention. Notably, the coefficient for pull factors was almost double that of push factors, pointing to a stronger effect size in shaping students’ migration decisions.

## 4. Discussion

The findings of this study highlight a strong inclination toward migration among medical and nursing students in Albania, primarily driven by pull factors such as better financial opportunities, job security, and improved living standards. The preference for migration observed in this study aligns with global trends, as medical professionals from lower-income countries often seek employment in developed nations due to better working conditions and career advancement opportunities. Similar patterns were observed in Pakistan, where financial incentives and career development were the primary motivators for physician migration [1].

The results indicate that medical students have significantly higher migration tendencies compared to nursing and midwifery students (*p* = 0.002). This is consistent with the study by Kansal et al. (2023) [2], which found that medical students, particularly those in their final years, demonstrated a stronger desire to pursue postgraduate training abroad. Likewise, research from Romania reported that medical students view international migration as an essential step in professional growth [11]. This suggests that specialized medical education is associated with a higher perceived value in international job markets, making migration a more attractive option. Moreover, in a study conducted in the Western Balkans, similar findings emerged, highlighting that students perceive migration as the best way to access higher salaries, modern healthcare systems, and better working conditions compared to their home country [12].

Another key finding is that third-year students (*p* = 0.011) and female students (*p* = 0.049) demonstrated higher migration tendencies. The tendency of students in their final undergraduate years to express stronger migration intentions aligns with research conducted in Kenya, which found that students nearing graduation were more likely to seek employment abroad due to job insecurity in their home country [13]. Additionally, the gender disparity observed in this study may be attributed to perceived workplace inequalities and professional barriers that female healthcare professionals face domestically, as noted in studies conducted in Eastern European countries [12]. Research from Albania has also indicated that young professionals, particularly women, often migrate due to limited career advancement opportunities and social constraints that hinder professional growth [3,14]. The role of push factors, while secondary to pull factors, is still significant. Political instability and concerns about safety and freedom were among the leading reasons why students considered migration, with 42.1% expressing a desire to live in a country with greater freedom of thought and personal security. This finding supports the argument that physicians from Eastern European countries frequently migrate due to dissatisfaction with governmental policies and political constraints in their home countries [15,16]. Furthermore, similar concerns were reported by Tariq et al. (2023) in their study on medical students in Pakistan, where political instability and lack of trust in the healthcare system contributed significantly to their intention to migrate [17].

The Albanian context is particularly relevant within the broader Western Balkan migration phenomenon, where young professionals often leave due to low salaries and limited career growth opportunities [14]. In line with findings from North Macedonia, the current study further supports that economic incentives remain the most influential driver of migration, surpassing political or social concerns [18]. Additionally, Silvestri et al. (2014) found similar migration patterns among medical students in Asia and Africa, where financial constraints and poor working conditions were leading reasons for seeking employment abroad [19].

The logistic regression findings offer empirical support for the push–pull theoretical framework in explaining emigration intentions among medical and nursing students in Albania. Both push and pull dimensions were strongly associated with the likelihood of intending to migrate, affirming the dual influence of dissatisfaction at home and attraction abroad.

Importantly, the pull model demonstrated a higher effect size, suggesting that aspirational drivers—such as the expectation of better salaries, working conditions, and postgraduate opportunities—may be more influential than push factors alone. This aligns with previous studies in similar regional contexts, which have found that migration decisions are increasingly driven by positive expectations rather than solely by local frustrations [4].

These results highlight the urgent need for policy responses that go beyond merely reducing dissatisfaction within the local system. Efforts to retain future health professionals should also include positive incentives, such as offering structured career paths, scholarships for specialization, and international collaboration that allows mobility without permanent loss.

Finally, while this study separately modeled push and pull effects, future research using more complex regression models should explore their joint contribution, potential interaction effects, and differential influence across nursing and medical student subgroups.

These results have important policy implications. The migration of nurses, in particular, poses an added threat to Albania’s healthcare system, where nursing workforce density is already among the lowest in Europe. If outbound migration trends continue, nurse shortages will likely intensify, compromising service delivery, especially in primary care and rural areas. A lack of local opportunities for specialization, career development, and professional recognition are key drivers pushing nurses abroad. Given the high migration intentions among medical students, Albanian policymakers should consider strategies to improve working conditions, offer competitive salaries, and create more career development opportunities within the country to retain healthcare professionals. Initiatives such as structured residency programs, financial incentives for medical professionals, and institutional reforms addressing workplace dissatisfaction may help mitigate the effects of brain drain, as suggested by Silvestri et al. (2014) [19]. Other countries, such as Romania have implemented retention strategies that include salary adjustments, medical education reforms, and improved job conditions, which could serve as a model for Albania [11].

Furthermore, the role of international cooperation in addressing brain drain cannot be overlooked. Several European countries have adopted policies aimed at reducing medical brain drain through bilateral agreements, educational exchange programs, and incentives for healthcare professionals to return after acquiring international experience [15]. Implementing similar strategies in Albania could contribute to sustaining a healthcare workforce while also allowing professionals to benefit from global medical advancements.

## 5. Limitations

The study used a self-report tool without in-depth qualitative data. While BDAS is validated, it does not decompose migration intent into economic vs. professional variables. This study did not include macro-level structural indicators of brain drain, such as national emigration rates or education-based opportunity gaps, which are often used in demographic and policy analyses. Future research may consider integrating these structural metrics—such as the degree of openness and education gap between emigrants and natives—to offer a more comprehensive multilevel perspective.

## 6. Future Research

Further research should examine longitudinal trends in migration intentions, evaluate institutional retention strategies, and compare findings across regions or professions.

## 7. Conclusions

In summary, this study confirms that migration tendencies among medical and nursing students in Albania are predominantly influenced by pull factors related to better financial prospects and professional growth abroad, with medical students and third-year students showing the highest migration inclination. These findings are consistent with trends observed in other developing regions, emphasizing the global nature of brain drain in healthcare. Addressing these challenges requires targeted policy interventions to improve retention and support for medical professionals in Albania, ensuring a sustainable healthcare workforce for the future. Collaborative efforts between government institutions, educational entities, and international organizations could play a crucial role in reducing migration rates and strengthening Albania’s healthcare system in the long term.

### Policy Recommendations

Establish competitive salary structures and structured postgraduate training programs to retain healthcare graduates, with targeted incentives for nurses who are at higher risk of early emigration.

Introduce mentorship and career development initiatives within nursing schools, including leadership training and international exchange opportunities, to improve job satisfaction and reduce migration intent among nursing students.

## Figures and Tables

**Table 1:** Demographic and educational characteristics of the study Participants.

Variables	N	%
Gender		
Female	532	87.2
Male	78	12.8
Age group		
18–20 years old	434	71.2
21–30 years old	149	24.4
>30 years old	27	4.4
Marital status		
Single	549	90.0
Married	58	9.5
Widowed	3	0.5
Field of study		
Nursing	487	79.8
Midwifery Nursing	73	12.0
Medicine	50	8.2
Level of Study		
Bachelor	569	93.3
Master	26	4.3
Residency	15	2.4
Year of Study		
First-year	259	42.5
Second-year	166	27.2
Third-year	166	27.2
Fourth-year	8	1.3
Fifth-year	11	1.8

**Table 2:** Mean scores on the brain drain attitude scale by demographic and educational characteristics of the participants.

Variables	Pull Factors	*p*	Push Factors	*p*	Total	*p*
Gender Female Male	40.40 ± 12.56 39.20 ± 13.96	Z ^†^ = −1.098 0.272	13.24 ± 4.04 12.85 ± 4.64	Z = −1.391 0.164	53.64 ± 16.60 52.05 ± 18.60	Z = −1.185 0.236
Age group 18–20 years old 21–30 years old >30 years old	40.20 ± 12.65 41.51 ± 12.93 39.48 ± 13.41	H ^§^ = 0.886 0.641	13.10 ± 4.14 13.40 ± 4.09 13.48 ± 3.99	H = 2.851 0.232	53.30 ± 16.79 53.90 ± 17.02 52.96 ± 17.40	H = 0.950 0.621
Marital status Single Married Widowed	40.42 ± 12.62 38.41 ± 14.01 43.67 ± 11.15	H = 1.982 0.370	13.18 ± 4.09 13.19 ± 4.49 14.33 ± 4.46	H = 1.057 0.582	53.60 ± 16.70 51.60 ± 18.50 58.00 ± 15.61	H = 1.447 0.484
Field of study Nursing Midwifery-Nursing Medicine	39.81 ± 12.73 41.23 ± 11.67 43.12 ± 13.74	H = 11.765 0.002	13.15 ± 4.17 13.04 ± 3.79 13.82 ± 4.12	H = 5.503 0.059	52.95 ± 16.90 54.27 ± 15.46 56.94 ± 17.86	H = 10.715 0.004
Level of Study Bachelor Master Residency	40.14 ± 12.67 42.96 ± 11.42 39.67 ± 16.96	H = 2.701 0.258	13.16 ± 4.13 13.92 ± 3.32 13.07 ± 5.01	H = 2.606 0.263	53.30 ± 16.80 56.88 ± 14.74 52.73 ± 21.97	H = 2.758 0.251
Year of Study First-year Second-year Third-year Fourth-year Fifth-year	39.45 ± 13.28 40.20 ± 12.63 41.22 ± 11.97 43.75 ± 11.59 42.55 ± 12.62	H = 7.581 0.107	13.05 ± 4.26 13.15 ± 4.16 13.42 ± 3.89 13.25 ± 3.60 13.64 ± 3.69	H = 2.908 0.562	52.49 ± 17.54 53.34 ± 16.78 54.65 ± 15.86 57.00 ± 15.19 56.18 ± 16.31	H = 6.616 0.151
Total points Mean totals	40.25 ± 12.76 3.30 ± 1.03		13.19 ± 4.13 3.30 ± 1.03		53.43 ± 16.88 3.34 ± 0.58	

Note: ^†^ Z = Mann–Whitney U test statistic; ^§^ H = Kruskal–Wallis test statistic; *p* < 0.05 was considered statistically significant.

## Data Availability

The raw data supporting the conclusions of this article will be made available by the authors upon request.

## Public Involvement Statement

No public involvement in any aspect of this research.

## Guidelines and Standards Statement

This manuscript was drafted against the STROBE guidelines for observational research. A complete list of reporting guidelines can be accessed via the equator network: https://www.strobe-statement.org/.

## Abbreviations

The following abbreviations are used in this manuscript:

BDAS	Brain Drain Attitude Scale
BD-s	Brain Drain score (abbreviated form used in-text for BDAS score)
SPSS	Statistical Package for the Social Sciences
CI	Confidence Interval
WHO	World Health Organization Questionnaire
